# Association of immunologic markers from complete blood counts with the response to preoperative chemoradiotherapy and prognosis in locally advanced rectal cancer

**DOI:** 10.18632/oncotarget.15760

**Published:** 2017-02-27

**Authors:** Sung Woo Jung, In Ja Park, Se Heon Oh, Seung-Seop Yeom, Jong Lyul Lee, Yong Sik Yoon, Chan Wook Kim, Seok-Byung Lim, Jung Bok Lee, Chang Sik Yu, Jin Cheon Kim

**Affiliations:** ^1^ Department of Colon and Rectal Surgery, University of Ulsan College of Medicine and Asan Medical Center, Seoul, Korea; ^2^ Department of Clinical Epidemiology and Biostatistics, University of Ulsan College of Medicine and Asan Medical Center, Seoul, Korea

**Keywords:** rectal cancer, preoperative chemoradiotherapy, neutrophil to lymphocyte ratio, tumor response, oncologic outcome

## Abstract

We investigated retrospectively whether immunologic markers from a complete blood count (CBC) are associated with the responsiveness to preoperative chemoradiotherapy (PCRT) and oncologic outcomes in 984 patients with locally advanced rectal cancer (LARC) who also underwent radical surgery from 2005 to 2013. CBC parameters including the neutrophil to lymphocyte ratio (NLR), lymphocyte to monocyte ratio (LMR), and platelet to lymphocyte ratio (PLR) were recorded. Pathologic responses to PCRT were evaluated in the resected specimens using the tumor regression grade system. The cut-off values of the immunologic markers were calculated to analyze their association with recurrence-free survival (RFS). One hundred ninety-five patients achieved total regression of their primary tumor. By receiver operating characteristic analysis, NLR, PLR, and LMR could not distinguish total regression from residual disease after PCRT. The NLR, LMR and PLR cut-off values were 1.7, 6.8 and 92.88, respectively. By univariate analysis, low NLR (≤1.7), high LMR (>6.8) and high PLR (>92.88) were indicators of a favorable RFS outcome. By multivariate analysis, high PLR was associated with an improved RFS (HR, 0.649; 95% CI, 0.473-0.89; *P*=0.007). High NLR (>1.7) was an independent negative prognostic factor for RFS in stage II (HR, 1.868; 95% CI, 1.08-3.109; *P*=0.025) and high PLR was a positive prognostic factor in stage III (HR, 0.675; 95% CI, 0.421-0.957; *P*=0.03). Immunologic markers derived from CBCs are independently associated with the RFS outcome in LARC patients treated with PCRT followed by radical resection. However, these markers are not predictive of total primary tumor regression after PCRT.

## INTRODUCTION

It is important to identify patients with colorectal cancer who are at risk of a poor outcome in order to better optimize the treatment approach. There has been increasing interest in improving prognostication in patients with colorectal cancer by developing better clinical, immunologic, and molecular biomarkers. It has been increasingly reported that inflammation is involved in the development of cancer and that an ongoing systemic inflammatory response is associated with a poorer prognosis in various types of cancer [1]. Moreover, the systemic inflammatory response is a known prognostic indicator in patients with colorectal cancer [2, 3]. Complete blood count (CBC)-based immunologic markers associated with inflammation have been evaluated as a cost-effective assessment of the relationship between prognosis and systemic inflammatory response. There are a number of studies demonstrating that the NLR (neutrophil to lymphocyte ratio), LMR (lymphocyte to monocyte ratio), and PLR (platelet to lymphocyte ratio), which are considered to reflect the host inflammatory and immunologic status, are prognostic factors in colorectal cancer [4–6].

Preoperative chemoradiotherapy (PCRT) is one of the standard treatment options for locally advanced rectal cancer (LARC, T3-4 and/or N1-2) and it has been reported that the resulting long-term oncologic outcomes of this therapy differ in terms of the tumor responsiveness [7]. There have been many reports of the various methods used to evaluate the prognosis and predict the response level with PCRT [8–10]. Similar to the results of patients who did not receive PCRT, some reports have indicated that these markers also have an association with the prognosis in patients treated with PCRT [5, 11, 12]. In addition, the possibility of using immunologic markers from CBCs as predictive markers for the treatment responsiveness to PCRT has also been suggested [13, 14]. However, the findings of these previous reports have been somewhat controversial and they have been small-scale studies only.

In our current study, we have further evaluated whether immunologic markers derived from CBCs are indeed associated with the treatment responsiveness to PCRT or with the oncologic outcomes in patients with locally advanced rectal cancer treated with PCRT followed by radical resection.

## RESULTS

### Patient characteristics and determination of the cut-off point

The clinicopathologic characteristics of the study patients are detailed in Table 1. Sixty-five percent of these patients were male, and the median age of 59 years (range, 26-86). Among these, 183 (18.6%) patients achieved total tumor regression (ypT0N0) and 223 (22.7%) developed local and/or distant recurrence. The median follow-up was 48 months (range 3-107).

**Table 1:** Clinicopathological characteristics of the study patients (*n*=984)

Variable	Value
**Age, median (range)**	59 (26-86)
**Gender**	
Male	640 (65%)
Female	344 (35%)
**ypT stage** **ypT0** **ypTis** **ypT1** **ypT2** **ypT3** **ypT4**	195 (19.8%) 15 (1.5%) 58 (5.9%) 274 (27.8%) 432 (43.9%) 10 (1.0%)
**ypN stage** **ypN0** **ypN1** **ypN2**	728 (74%) 194 (19.7%) 62(6.3%)
**Histologic differentiation**	
Well, moderately	942 (95.7%)
Poorly, mucinous, signet ring cell	42 (4.3%)
**Lymphovascular invasion**	84 (9.5%)
**Perineural invasion**	117 (11.9%)
**Tumor regression grade of primary tumor**	
Total	195 (19.8%)
Near total	220 (22.4%)
Moderate	418 (42.5%)
Minimal & no	151 (15.3%)

The Contal and Q`Quigley method was used to find the optimal cut-off point for the NLR, LMR and PLR values. In the analysis of the total patient population, a cut-off of 1.7 for the NLR was found to have the highest log-rank statistic of any cut-off value. We subsequently categorized patients into low NLR (≤1.7) and high NLR ( > 1.7) groups. For the LMR and PLR values, cut-off points of 6.8 and 92.88 were identified, respectively and high and low groups were established accordingly. Patient characteristics based on NLR, LMR, and PLR cut-off groupings were then compared. In the high NLR, low LMR and low PLR groups, male patients were predominant (*P* < 0.001). Patients in the high NLR group also tended to have a more advanced ypT stage (*P* = 0.014) and ypN1 stage (*P* = 0.003) whereas patients in the low LMR group were associated with a higher ypT stage (*P* = 0.04; Supplementary Tables 1-3).

**Table 3:** Univariate and multivariate analysis of factors associated with recurrence-free survival

Variables	Univariate		Multivariate		
	Hazard ratio	*P*	Hazard ratio	95% CI	*P*
**NLR**		0.008			0.07
Low NLR (≤1.7) High NLR (>1.7)	1 1.446		1 1.319	0.978-5.087	
**LMR** Low LMR (≤6.8) High LMR (>6.8)	1 0.53	0.008	1 0.658	0.405-1.070	0.092
**PLR** Low PLR (≤92.88) High PLR (>92.88)	1 0.765	0.076	1 0.649	0.473-0.89	0.007
**ypT stage**		<0.001			<0.001
ypT0	1		1		
ypT1 ypT2 ypT3 ypT4	0.388 1.852 5.216 6.235		0.417 1.694 3.297 3.208	0.097-1.799 0.972-1.449 1.980-5.491 0.74-14.7	
**ypN stage** ypN0 ypN1 ypN2	1 2.931 5.347	<0.001	1 1.882 2.345	1.385-2.557 1.538-3.575	<0.001
**Lymphovascular invasion**	3.637	<0.001	1.762	1.229-2.526	0.002
**Perineural invasion**	3.195	<0.001	1.509	1.093-2.083	0.012
**Age, yrs** ≤70 >70	1 1.09	0.648			
Gender Male Female	1 0.893	0.431			

NLR, neutrophil to lymphocyte ratio; LMR, lymphocyte to monocyte ratio; PLR, platelet to lymphocyte ratio

### Association between immunologic markers and pathologic tumor regression

The NLR was found to be strongly associated with both the LMR (*P* < 0.001) and PLR (*P* < 0.001; Table 2). Specifically, a high NLR was more likely in the low LMR group (61.1%) than in the high LMR group (21,4%). Similarly, a high PLR was more common in patients with a low NLR (61%) than in those with a high NLR (9.6%) and a high LMR was more common in patients with low PLR (88.2 %) than in those with high PLR (11.8%; Table 2). The proportion of patients who showed total regression was not significantly different in the NLR, LMR, and PLR groups (Supplementary Tables 1-3). In the ROC analysis, the AUC (area under the curve) for the NLR, PLR, and LMR was 0.55 (*P* = 0.04), 0.49 (*P* = 0.62) and 0.54 (*P* = 0.13) for tumor responsiveness, respectively. Therefore, it was confirmed that these markers could not be used to distinguish total regression from the residual disease.

**Table 2:** Distribution of NLR, LMR, and PLR values (%)

NLR vs. LMR, *P*< 0.001			
	**Low LMR (≤6.8)**		**High LMR (>6.8)**
Low NLR (≤1.7)	327 (74.7)		111 (25.3)
High NLR (>1.7)	516 (94.5)		30 (5.5)
**NLR** ***vs*. PLR,** ***P*** **< 0.001**			
	**Low PLR (≤92.88)**	**High PLR (>92.88)**	
Low NLR (≤1.7)	170 (38.8)	268 (61.2)	
High NLR (>1.7)	52 (9.5)	494 (90.5)	
**LMR** ***vs*. PLR,** ***P*** **< 0.001**			
	**Low PLR (≤92.88)**	**High PLR (>92.88)**	
Low LMR (≤6.8)	170 (20.2)	663 (79.8)	
High LMR (>6.8)	52 (36.9)		89 (63.1)

NLR, neutrophil to lymphocyte ratio; LMR, lymphocyte to monocyte ratio; PLR, platelet to lymphocyte ratio

### Association between immunologic markers and RFS

To determine whether any association existed between clinicopathologic factors including immunologic markers and RFS outcomes, univariate analyses were performed (Table 3). The ypT stage, ypN stage, lymphovascular invasion, perineural invasion, NLR, and LMR showed a significant association with RFS. The PLR tended to be related to the RFS outcome. The significant variables were then evaluated by multivariate analyses (Table 3) which showed that a high PLR was associated with a better RFS (hazard ratio 0.649, 95% confidence interval: 0.473-0.89, *P* = 0.007), independently of the ypT stage (*P* < 0.001), ypN stage (*P* < 0.001), lymphovascular invasion (*P* = 0.002), or perineural invasion (*P* = 0.012).

**Figure 1:**
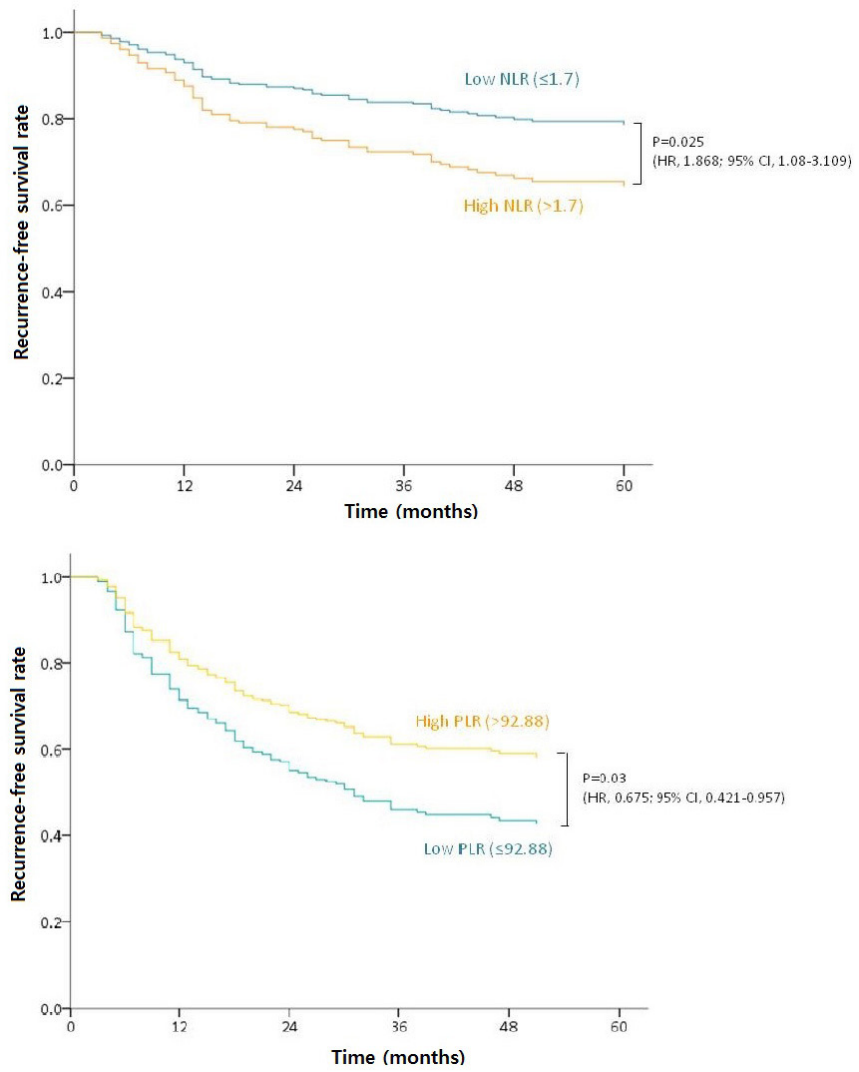
RFS outcomes in patients with different cancer stages **A**. RFS according to high and low NLR in ypstage II patients. **B**. A high PLR was associated with a better RFS in ypstage III cases.

**Figure 2:**
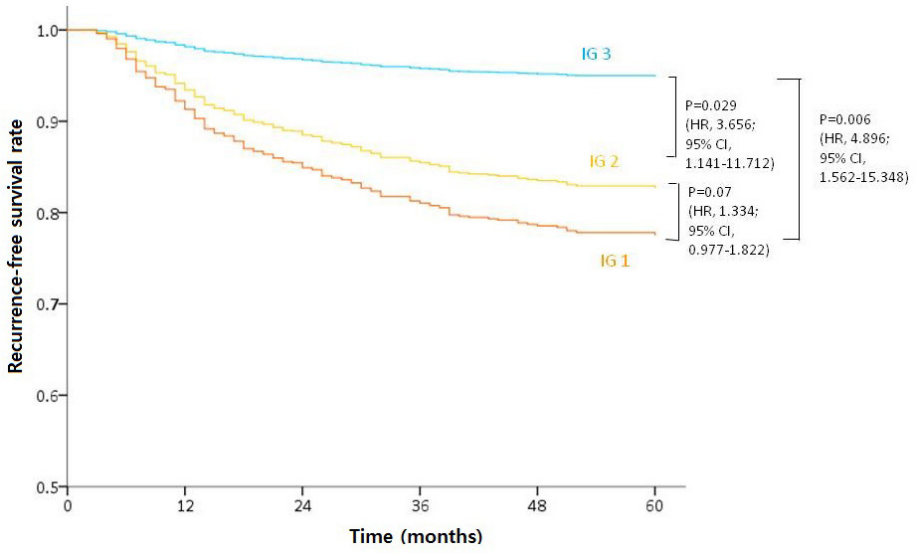
RFS according to immunologic groupings of a low NLR, high LMR, and PLR IG3 patients showed a significantly better RFS rate regardless of other prognostic factors.

In a separate analysis of individual cancer stages, NLR was found by multivariate analysis to be independently prognostic for stage II (*P* = 0.025) and the PLR to be associated with RFS in stage III (*P* = 0.03). However, none of the NLR, LMR, or PLR values were independently associated with stage I disease (Figure 1). Cox regression analysis further indicated that a low NLR, high LMR, and high PLR were indicators of a favorable RFS. Considering these characteristics of these ratios together, we categorized patients into different immunologic groups (IG). Patients without any favorable factor or only 1 favorable factor were categorized as IG1, those with 2 favorable factors as IG2, and those with all 3 favorable factors as IG3. By multivariate analysis, IG was confirmed as an independent prognostic factor for RFS regardless of pathologic stage, lymphovascular invasion, or perineural invasion (Table 4). IG3 patients showed significantly better RFS outcomes than either IG1 or 2 (Figure 2).

**Table 4:** Association between immunologic groups and recurrence-free survival by multivariate analysis

Variables	Hazard ratio	95% CI	*P*
**Immunologic group (IG)**			0.006
IG1 IG2 IG3	1 3.656 4.896	1.141-11.712 1.562-15.348	
**yp stage**			<0.001
Complete remission	1		
yp stage I yp stage II yp stage III	1.544 3.847 6.059	0.802-2.971 2.116-6.994 3.353-10.949	
**Lymphovascular invasion**	1.968	1.389-2.789	<0.001
**Perineural invasion**	1.601	1.164-2.202	0.004

## DISCUSSION

We find from our current analyses that immunologic markers derived from CBCs are associated with the RFS outcomes in patients with locally advanced rectal cancer who had been treated with PCRT and radical resection. PLR was found to be an independent prognostic factor for the RFS. However, none of the CBC immunologic markers could predict or be associated with total regression of the primary tumor after PCRT. Considering these immunologic markers together, patients who with favorable NLR, LMR, and PLR values showed a significantly better RFS outcome than other patients with 1 or 2 favorable markers. We also evaluated whether the CBC immunologic marker profile is predictive of the response to PCRT and simultaneously evaluated its relationship with RFS outcomes in a large rectal cancer population. We assessed whether the immunologic factors prior to PCRT as well as pathologic risk factors after this therapy influenced the oncologic outcome. Because the change in the primary tumor status after PCRT was an important component of the prognosis, we evaluated whether the pretreatment immunologic factor ratios could affect this.

There have been many suggestions that inflammatory and immunologic markers derived from a CBC are associated with cancer development and progression. Cytokines, growth factors, proteases, and other cellular mediators are secreted stemming from tumor and flow into the systemic circulation where they exert both local and systemic inflammatory effects. One particular effect involves changes to the hematologic system [15–17]. Lymphocytes are known to play an important role in the development and progression of cancer through the regulation of cell-mediated immunity. Cancer cells secrete anti-inflammatory cytokines such as IL-4, IL-10, and TGF-β and induce systemic inflammation and suppress lymphocyte function. Neutrophils are known to secrete angiogenic chemokines and contribute to angiogenesis during cancer development [18–20]. Platelet and tumor-associated macrophages derived from circulating monocytes play an important role in the tumor microenvironment and are known to affect tumor growth and metastasis [5, 21]. Thus, some studies have reported that that NLR, PLR, and LMR values are associated with the prognosis in colorectal cancer patients [5, 22, 23]. These markers would, therefore, reflect inflammatory processes at both the tumor level and also systemically.

Several investigators have previously reported that immunologic markers are associated with the prognosis after PCRT in LARC patients. Although the cutoff values reported in each study were different, several retrospective studies described that a low NLR in patients who underwent surgery after PCRT was associated with a poorer prognosis [11–13]. Joseph et al. [5] published that the LMR was a superior independent predictor of OS in patients who underwent curative resection for colorectal cancer. Similar results were obtained in our present study. We found in our LARC patient series that a low NLR, high LMR, and high PLR were favorable indicators for the RFS outcome after PCRT and surgery. We found in particular by multivariate analysis that a high PLR was a statistically significant prognostic factor in this regard. In contrast to other studies that analyzed each marker separately, our current study showed that the number of favorable markers may be significantly associated with a positive RFS outcome.

In previous studies of the preoperative PLR and oncologic outcome in patients with colorectal cancer, some investigators have claimed that the prognosis of patients with a high PLR was poorer, whilst others reported that the relationship between PLR and the surgical outcome was unclear [6, 24–26]. In contrast, we here studied a relatively large cohort of patients who underwent PCRT for rectal cancer and found that a high PLR after this therapy was an independent predictor of a better RFS after surgery in these patients. Studies of the changes in the immunological status related to the PLR of patients after PCRT will be needed in the future, and another large-scale study of the relationship between the PLR and oncologic outcome after PCRT may confirm this variable as a definitive and independent prognostic factor in colorectal cancer.

Several studies have suggested that the NLR is a predictor of pathologic tumor regression in rectal cancer patients after PCRT [1, 13, 14], whereas our current findings using ROC analysis of the AUC of tumor response prediction indicate that the NLR, PLR, and LMR do not correlate with the total response to PCRT. Further studies are thus required to evaluate whether these markers can be used for predicting the tumor response to PCRT.

Although our present study involved a large sample size, our findings were limited by the retrospective nature of our analyses. Compared to the total number of patients, the cancer stage subgroups were not very large when analyzing associations. To fully determine the utility of immunologic markers as valuable prognostic indicators, a well-designed prospective study is needed.

Another notable limitation of our current study was that if the immunologic marker value changed due to inflammatory events such as infection or trauma at the time of blood sampling, the normal immune status of the patient might not have been fully reflected in the results. Also, because of retrospective studies, it was possible that patients with unrecorded infectious diseases were included in this study.

Variable cut-off points of immunologic markers are one of the limitations of this study. Since cut-off points in this and other previous studies analyzing immunologic markers of patients with LARC are not constant, we believe that another large-scale study or prospective study should confirm accurate cut-off points. Despite these limitations, we set up immunologic groups (IG) by combining cut-off points of NLR, LMR, and PLR in this study, have found that these are related to prognosis of patients with LARC.

In summary, and notwithstanding the abovementioned limitations, we have studied a considerable number of well-controlled colorectal cancer patients that had undergone PCRT with surgery, and their TRGs were confirmed by highly specialized pathologists, minimizing the errors that could have affected our results. We found that a low NLR, high LMR, and high PLR could be favorable factors for the RFS and analyzed the effects of these three variables on the patient prognosis. Importantly, none of the three CBC immunologic markers (NLR, PLR, and LMR) were found to be predictive of total regression of the primary tumor after PCRT in our LARC patients. Of these three markers, PLR was found to be an independent predictor of the RFS outcome after PCRT and surgery. Moreover, a low NLR, high LMR, and high PLR were favorable factors for the RFS and patients with more of these factors showed a better RFS outcome.

## MATERIALS AND METHODS

### Study patients

A retrospective cohort of patients with biopsy-proven, locally advanced rectal cancer (cT3-4 and/or cN1-2) who underwent PCRT followed by total mesorectal excision (TME) at Asan Medical Center between October 2005 and December 2013 was analyzed. Magnetic resonance imaging (MRI), or transrectal ultrasound (TUS) was used to determine the pretreatment local clinical stage in these cases. Among the patients identified from our institutional colorectal cancer patient registry, 984 patients who underwent PCRT followed by TME were finally enrolled in this study. Retrievable pretreatment blood samples were obtained within 7 days before the start of PCRT. The white blood cell count (WBC), neutrophil count, lymphocyte count and platelet count were recorded. The NLR, LMR, and PLR were then calculated as systemic immunologic markers. Patients with the acute infectious condition were excluded. This study was approved by the Institutional Review Board of Asan Medical Center. (Registration no: 2016-1022).

### Preoperative chemoradiotherapy, surgery, and pathologic examination

Preoperative radiotherapy consisted of 25 fractions at a dosage of 45-50 Gy administered to the entire pelvis, followed by a 5.4-Gy boost to the primary tumor administered in 3 fractions. Chemotherapy was delivered in 2 cycles *via* an intravenous bolus of 5-fluorouracil (375 mg/m^2^/day) and leucovorin (20 mg/m^2^/day) over 3 days during the first and fifth weeks of radiation therapy, or *via* oral capecitabine (1650 mg/m^2^/day) twice-daily during the period of radiation therapy. Radical surgical resection was planned for 6-8 weeks after completing PCRT. Surgical resection was performed according to the principle of total mesorectal excision. The pathologic stage (ypT and ypN) was recorded according to the 7^th^ edition of the TNM classification of the American Joint Committee on Cancer (AJCC) [27]. Pathologic responses to PCRT were evaluated in the resected specimens using the tumor regression grade (TRG) system suggested by the Gastrointestinal Pathology Study Group of the Korean Society of Pathologists [28]. Patients were categorized into total regression and residual disease groups.

### Follow-up and oncologic outcomes

All patients received postoperative follow-up examinations, which consisted of a physical examination, serum carcinoembryonic antigen measurement, chest radiography, and abdominal, pelvic, and chest computed tomography every 3-6 months. Most patients underwent a colonoscopy at 6 months to 1 year postoperatively, and every 2-3 years thereafter. Recurrence was determined according to radiological or histopathological findings. Local recurrence was defined as the presence of a suspicious lesion in an area contiguous to the bed of the primary rectal resection or the site of anastomosis, and distant metastasis was defined as the presence of any recurrence in a distant organ or dissemination to the peritoneal surface. Recurrence-free survival (RFS) was measured from the date of surgery to the date of the first recurrence event or death.

### Statistical analysis

The clinical characteristics of the study patients were compared using the Pearson's chi-square test, Fisher exact test, or student *t* test, as applicable. The log-rank test using the Contal and Q`Quigley method was utilized to calculate the optimal cut-off for the immunologic markers for RFS. Receiver operating characteristic (ROC) analysis and relative area under the curve (AUC) statistics were used to select the optimal cut-off value to predict total regression after PCRT. The Kaplan-Meier method was used to calculate RFS outcomes in the study groups followed by comparisons using the log-rank test. Associations between clinical and immunologic markers (NLR, MLR, and PLR) and RFS were summarized as hazard ratios (HR) and 95% confidence intervals (CI) using Cox proportional hazards regression analysis. Statistical significance was defined as a *P* < 0.05, and all analyses were performed using SPSS software, version 21.0 (IBM Statistics, Armonk, NY).

## SUPPLEMENTARY MATERIALS FIGURES AND TABLES



## Abbreviations

CBC: complete blood count
WBC: white blood cell count
NLR: neutrophil to lymphocyte ratio
LMR: lymphocyte to monocyte ratio
PLR: platelet to lymphocyte ratio
PCRT: preoperative chemoradiotherapy
LARC: locally advanced rectal cancer
TME: total mesorectal excision
MRI: Magnetic resonance imaging
TUS: transrectal ultrasound
AJCC: the American Joint Committee on Cancer
RFS: Recurrence-free survival
ROC: Receiver operating characteristic
AUC: area under the curve
HR: hazard ratios
CI: confidence intervals
IG: immunologic groups
LVI: lymphovascular invasion
PNI: perineural invasion
WD: well-differentiated
MD: moderate-differentiated
PD: poorly-differentiated;
SRC: signet ring cell carcinoma
MUC: mucinous carcinoma
