# Supplementary material for: Association of immunologic markers from complete blood counts with the response to preoperative chemoradiotherapy and prognosis in locally advanced rectal cancer
Source: Oncotarget. 2017 Feb 27;8(35):59757–65. doi: 10.18632/oncotarget.15760 (PMC5601775; doi:10.18632/oncotarget.15760)
Supplement: Supplementary file 1 [file oncotarget-08-59757-s001.pdf]

Association of immunologic markers from complete blood counts with the response to preoperative chemoradiotherapy and prognosis in locally advanced rectal cancer

Supplementary Material

Supplementary Table 1: Clinicopathologic characteristics of the study patients based on NLR 1.7 (n=984)

Variable		n (%)	Low NLR(%) n=438 (44.5)	High NLR(%) n=546 (55.5)	*P*-value
Age, median (range)			59 (29-83)	59 (26-86)	0.49
Gender	Male	640 (65.0)	261 (59.6)	379 (69.4)	<0.001
	Female	344 (35.0)	177 (40.4)	167 (30.6)	
ypT stage	T0	195 (19.8)	97 (22.1)	98 (17.9)	0.014
	Tis	15 (1.5)	3 (0.7)	12 (2.2)	
	T1	58 (5.9)	29 (6.6)	29 (5.3)	
	T2	274 (27.8)	136 (31.1)	138 (25.3)	
	T3	432 (43.9)	170 (38.8)	262 (48.0)	
	T4	10 (1.0)	3 (0.7)	7 (1.3)	
ypN stage	N0	728 (74.0)	344 (78.5)	384 (70.3)	0.003
	N1	194 (19.7)	65 (14.8)	129 (23.6)	
	N2	62 (6.3)	29 (6.6)	33 (6.0)	
ypStage	0	198 (20.1)	98 (22.4)	100 (18.3)	<0.001
	I	275 (27.9)	143 (32.6)	132 (24.2)	
	II	253 (25.7)	101 (23.1)	152 (27.8)	
	III	258 (26.2)	96 (21.9)	162 (29.7)	
Differentiation	WD+MD	933 (95.7)	414 (95.2)	519 (96.1)	0.53
	PD+SRC+MUC	42 (4.3)	21 (4.8)	21 (3.9)	
LVI	(-)	900 (91.5)	409 (93.4)	491 (89.9)	0.07
	(+)	84 (8.5)	23 (6.6)	55 (10.1)	
PNI	(-)	867 (88.1)	395 (90.2)	472 (86.4)	0.08
	(+)	117 (11.9)	43 (9.8)	74 (13.6)	
TRG	Total	195 (19.8)	97 (22.1)	98 (17.9)	0.25
	Near total	220 (22.4)	99 (22.6)	121 (22.2)	
	Moderate	418 42.5)	183 (41.8)	235 (43.0)	
	Minimal & no	151 (15.3)	59 (13.5)	92 (16.8)	

LVI, lymphovascular invasion; PNI, perineural invasion; TRG, tumor regression grade; WD, well-differentiated; MD, moderate-differentiated; PD, poorly-differentiated; SRC, signet ring cell carcinoma; MUC, mucinous carcinoma

Supplementary Table 2: Clinicopathologic characteristics of the study patients based on LMR 6.8 (n=984)

Variable		n (%)	Low LMR (%) n=843 (85.7)	High LMR(%) n=141 (14.3)	*P*-value
Age, median (range)			59 (26-86)	58 (29-80)	0.33
Gender	Male	640 (65.0)	577 (68.4)	63 (44.7)	<0.001
	Female	344 (35.0)	266 (31.6)	78 (55.3)	
ypT stage	T0	195 (19.8)	159 (18.9)	36 (25.5)	0.04
	Tis	15 (1.5)	14 (1.7)	1 (0.7)	
	T1	58 (5.9)	47 (5.6)	11 (7.8)	
	T2	274 (27.8)	227 (26.9)	47 (33.3)	
	T3	432 (43.9)	387 (45.9)	45 (31.9)	
	T4	10 (1.0)	9 (1.1)	1 (0.7)	
ypN stage	N0	728 (74.0)	618 (73.3)	110 (78.0)	0.49
	N1	194 (19.7)	171 (20.3)	23 (16.3)	
	N2	62 (6.3)	54 (6.4)	8 (5.7)	
ypStage	0	198 (20.1)	162 (19.2)	36 (25.5)	0.06
	I	275 (27.9)	229 (27.2)	46 (32.6)	
	II	253 (25.7)	227 (26.9)	26 (18.4)	
	III	258 (26.2)	225 (26.7)	33 (23.4)	
Differentiation	WD+MD	933 (95.7)	799 (95.7)	134 (95.7)	0.99
	PD+SRC+MUC	42 (4.3)	36 (4.3)	6 (4.3)	
LVI	(-)	900 (91.5)	767 (91.0)	133 (94.3)	0.25
	(+)	84 (8.5)	76 (9.0)	8 (5.7)	
PNI	(-)	867 (88.1)	739 (87.7)	128 (90.8)	0.33
	(+)	117 (11.9)	104 (12.3)	13 (9.2)	
TRG	Total	195 (19.8)	159 (18.9)	36 (25.5)	0.3
	Near total	220 (22.4)	193 (22.9)	27 (19.1)	
	Moderate	418 42.5)	360 (42.7)	58 (41.1)	
	Minimal & no	151 (15.3)	131 (15.5)	20 (14.2)	

LVI, lymphovascular invasion; PNI, perineural invasion; TRG, tumor regression grade;
WD, well-differentiated; MD, moderate-differentiated; PD, poorly-differentiated;
SRC, signet ring cell carcinoma; MUC, mucinous carcinoma

Supplementary Table 3: Clinicopathologic characteristics of the study patients based on PLR 92.88 (n=984)

Variable		n (%)	Low PLR (%) n=222 (22.6)	High PLR (%) n=762 (77.4)	*P*-value
Age, median (range)			60 (29-81)	58 (26-86)	0.01
Gender	Male	640 (65.0)	170 (76.6)	470 (61.7)	<0.001
	Female	344 (35.0)	52 (23.4)	292 (38.3)	
ypT stage	T0	195 (19.8)	41 (18.5)	154 (20.2)	0.22
	Tis	15 (1.5)	0 (0.0)	15 (2.0)	
	T1	58 (5.9)	12 (5.4)	46 (6.0)	
	T2	274 (27.8)	70 (31.5)	204 (26.8)	
	T3	432 (43.9)	98 (44.1)	334 (43.8)	
	T4	10 (1.0)	1 (0.5)	9 (1.2)	
ypN stage	N0	728 (74.0)	160 (72.1)	568 (74.5)	0.29
	N1	194 (19.7)	43 (19.4)	151 (19.8)	
	N2	62 (6.3)	19 (8.6)	43 (5.6)	
ypStage	0	198 (20.1)	40 (18.0)	158 (20.7)	0.67
	I	275 (27.9)	65 (29.3)	210 (27.6)	
	II	253 (25.7)	54 (24.3)	199 (26.1)	
	III	258 (26.2)	63 (28.4)	195 (25.6)	
Differentiation	WD+MD	933 (95.7)	214 (97.3)	719 (95.2)	0.26
	PD+SRC+MUC	42 (4.3)	6 (2.7)	36 (4.8)	
LVI	(-)	900 (91.5)	206 (92.8)	694 (91.1)	0.5
	(+)	84 (8.5)	16 (7.2)	68 (8.9)	
PNI	(-)	867 (88.1)	197 (88.7)	670 (87.9)	0.81
	(+)	117 (11.9)	25 (11.3)	92 (12.1)	
TRG	Total	195 (19.8)	41 (18.5)	154 (20.2)	0.2
	Near total	220 (22.4)	43 (19.4)	177 (23.2)	
	Moderate	418 42.5)	108 (48.6)	310 (40.7)	
	Minimal & no	151 (15.3)	30 (13.5)	121 (15.9)	

LVI, lymphovascular invasion; PNI, perineural invasion; TRG, tumor regression grade; WD, well-differentiated; MD, moderate-differentiated; PD, poorly-differentiated; SRC, signet ring cell carcinoma; MUC, mucinous carcinoma.
